# Supplementary material for: Cutoff Values for Providing the Ideal Intravenous Patient-Controlled Analgesia According to the Intensity of Postoperative Pain—A Retrospective Observational Study
Source: Medicina (Kaunas). 2021 Oct 6;57(10):1065. doi: 10.3390/medicina57101065 (PMC8541173; doi:10.3390/medicina57101065)
Supplement: Supplementary file 1 [file medicina-57-01065-s001.zip › medicina-1373813-supplementary.pdf]

medicina

MDPI

Table S1. Cutoff values of potential variables for requiring rescue analgesics in patients with low PPI (n = 640)

Potential variables	Cutoff value	AUC	Sens. (%)	Spec. (%)	Youden index	95% CI	p value
PCA setting							
BIR (1 mL/h)	1.75	0.515	99.8	3.8	0.036	0.454, 0.577	0.627
Bolus volume (1 mL)	0.5	0.610	90.8	33.0	0.238	0.546, 0.674	0.001*
Lockout interval (min)	12.5	0.619	87.6	36.8	0.244	0.555, 0.682	<0.001*
Dose (μg)							
DOSE-FEN-OP†	950	0.559	48.7	69.8	0.185	0.505, 0.612	0.032*
DOSE-FEN-NONOP†	250	0.501	96.1	4.7	0.008	0.442, 0.561	0.971
DOSE-FEN-TOTAL†	1750	0.541	35.6	78.3	0.139	0.486, 0.597	0.143
BIR (μg/h)							
BIR-FEN-OP†	19	0.567	48.7	69.8	0.185	0.513, 0.622	0.015*
BIR-FEN-NONOP†	7	0.509	95.7	8.5	0.042	0.448, 0.569	0.783
BIR-EME	15	0.510	99.6	4.7	0.043	0.449, 0.571	0.756
BIR-FEN-TOTAL†	35	0.548	35.6	78.3	0.139	0.492, 0.605	0.095

AUC, area under the curve; BIR, background infusion rate; BIR-EME, BIR for adjuvant antiemetics; CI, confidence interval. Sens., Sensitivity; Spec., Specificity. *, statistical significance at $p < 0.05$. †, Potential variables (Doses and BIRs) were fentanyl equivalent (μg) doses converted for opioids (DOSE-FEN-OP), non-opioid adjuvant analgesics (DOSE-FEN-NONOP), and total analgesics (DOSE-FEN-TOTAL) using the ratios of oxycodone (μg) to fentanyl (100:1), sufentanil (μg) to fentanyl (1:10), ketorolac (mg) to fentanyl (25:100), and nefopam (mg) to fentanyl (20:100), and BIRs were recalculated with these converted doses (BIR-FEN-OP, BIR-FEN-NONOP, and BIR-FEN-TOTAL).

Table S2. Cutoff values of potential variables for requiring rescue analgesics in patients with moderate PPI (n = 2666)

Potential variables	Cutoff value	AUC	Sens. (%)	Spec. (%)	Youden index	95% CI	p value
PCA setting							
BIR (1 mL/h)	1.75	0.504	98.7	2.3	0.010	0.476, 0.531	0.790
Bolus volume (1 mL)	0.5	0.524	95.1	17.3	0.124	0.495, 0.552	0.104
Lockout interval (min)	5	0.512	92.1	18.4	0.105	0.483, 0.540	0.414
Dose (μg)							
DOSE-FEN-OP†	950	0.612	49.1	70.5	0.196	0.587, 0.638	<0.001*
DOSE-FEN-NONOP†	550	0.500	90.0	12.8	0.028	0.472, 0.528	0.986
DOSE-FEN-TOTAL†	1750	0.583	35.2	77.3	0.125	0.557, 0.610	<0.001*
BIR (μg/h)							
BIR-FEN-OP†	19	0.610	48.9	70.4	0.193	0.584, 0.635	<0.001*
BIR-FEN-NONOP†	11	0.500	89.2	13.9	0.031	0.473, 0.528	0.979
BIR-EME	21	0.504	92.7	8.3	0.010	0.477, 0.532	0.762
BIR-FEN-TOTAL†	35	0.581	35.1	77.1	0.122	0.555, 0.608	<0.001*

AUC, area under the curve; BIR, background infusion rate; BIR-EME, BIR for adjuvant antiemetics; CI, confidence interval. Sens., Sensitivity; Spec., Specificity. *, statistical significance at $p < 0.05$. †, Potential variables (Doses and BIRs) were fentanyl equivalent (μg) doses converted for opioids (DOSE-FEN-OP), non-opioid adjuvant analgesics (DOSE-FEN-NONOP), and total analgesics (DOSE-FEN-TOTAL) using the ratios of oxycodone (μg) to fentanyl (100:1), sufentanil (μg) to fentanyl (1:10), ketorolac (mg) to fentanyl (25:100), and nefopam (mg) to fentanyl (20:100), and BIRs were recalculated with these converted doses (BIR-FEN-OP, BIR-FEN-NONOP, and BIR-FEN-TOTAL).

Table S3. Cutoff values of potential variables for requiring rescue analgesics in patients with high PPI grade (n = 800)

Potential variables	Cutoff value	AUC	Sens. (%)	Spec. (%)	Youden index	95% CI	p value
PCA setting							
BIR (1 mL/h)	1.75	0.508	99.7	1.9	0.016	0.462, 0.553	0.741
Bolus volume (1 mL)	0.5	0.573	93.9	24.4	0.183	0.526, 0.620	0.002*
Lockout interval (min)	5	0.605	91.1	32.4	0.235	0.558, 0.652	<0.001*
Dose (μg)							
DOSE-FEN-OP†	950	0.660	54.2	74.6	0.288	0.617, 0.703	<0.001*
DOSE-FEN-NONOP†	700	0.540	64.4	41.8	0.062	0.494, 0.586	0.088
DOSE-FEN-TOTAL†	1550	0.656	70.9	58.7	0.296	0.612, 0.699	<0.001*
BIR (μg/h)							
BIR-FEN-OP†	19	0.662	54.0	74.6	0.286	0.619, 0.704	<0.001*
BIR-FEN-NONOP†	14	0.546	64.2	42.7	0.069	0.500, 0.592	0.050
BIR-EME	21	0.545	92.0	16.9	0.089	0.499, 0.592	0.057
BIR-FEN-TOTAL†	31	0.658	70.7	59.2	0.299	0.615, 0.701	<0.001*

AUC, area under the curve; BIR, background infusion rate; BIR-EME, BIR for adjuvant antiemetics; CI, confidence interval. Sens., Sensitivity; Spec., Specificity. *, statistical significance at $p < 0.05$. †, Potential variables (Doses and BIRs) were fentanyl equivalent (μg) doses converted for opioids (DOSE-FEN-OP), non-opioid adjuvant analgesics (DOSE-FEN-NONOP), and total analgesics (DOSE-FEN-TOTAL) using the ratios of oxycodone (μg) to fentanyl (100:1), sufentanil (μg) to fentanyl (1:10), ketorolac (mg) to fentanyl (25:100), and nefopam (mg) to fentanyl (20:100), and BIRs were recalculated with these converted doses (BIR-FEN-OP, BIR-FEN-NONOP, and BIR-FEN-TOTAL).

Table S4. Cutoff values of potential variables for requiring rescue antiemetics in patients with low PPI (n = 640)

Potential variables	Cutoff value	AUC	Sens. (%)	Spec. (%)	Youden index	95% CI	p value
PCA setting							
BIR (1 mL/h)	3	0.494	0.0	100.0	0.000	0.352, 0.635	0.929
Bolus volume (1 mL)	1.25	0.576	75.5	43.7	0.192	0.433, 0.720	0.297
Lockout interval (min)	17.5	0.583	69.6	50.0	0.196	0.443, 0.723	0.246
Dose (μg)							
DOSE-FEN-OP†	950	0.615	46.2	75.0	0.212	0.487, 0.744	0.079
DOSE-FEN-NONOP†	50	0.470	96.2	6.2	0.024	0.328, 0.612	0.679
DOSE-FEN-TOTAL†	1350	0.543	79.5	31.2	0.107	0.400, 0.686	0.554
BIR (μg/h)							
BIR-FEN-OP†	19	0.613	46.2	75.0	0.212	0.485, 0.740	0.083
BIR-FEN-NONOP†	1	0.468	96.2	6.2	0.024	0.327, 0.610	0.661
BIR-EME	25	0.474	0.00	100.0	0.000	0.337, 0.610	0.704
BIR-FEN-TOTAL†	27	0.541	79.2	31.2	0.104	0.400, 0.683	0.567

AUC, area under the curve; BIR, background infusion rate; BIR-EME, BIR for adjuvant antiemetics; CI, confidence interval. Sens., Sensitivity; Spec., Specificity. *, statistical significance at $p < 0.05$. †, Potential variables (Doses and BIRs) were fentanyl equivalent (μg) doses converted for opioids (DOSE-FEN-OP), non-opioid adjuvant analgesics (DOSE-FEN-NONOP), and total analgesics (DOSE-FEN-TOTAL) using the ratios of oxycodone (μg) to fentanyl (100:1), sufentanil (μg) to fentanyl (1:10), ketorolac (mg) to fentanyl (25:100), and nefopam (mg) to fentanyl (20:100), and BIRs were recalculated with these converted doses (BIR-FEN-OP, BIR-FEN-NONOP, and BIR-FEN-TOTAL).

Table S5. Cutoff values of potential variables for requiring rescue antiemetics in patients with moderate PPI (n = 2666)

Potential variables	Cutoff value	AUC	Sens. (%)	Spec. (%)	Youden index	95% CI	p value
PCA setting							
BIR (1 mL/h)	1.75	0.523	98.6	6.0	0.046	0.450, 0.596	0.534
Bolus volume (1 mL)	1.75	0.519	77.1	28.4	0.055	0.45, 0.588	0.592
Lockout interval (min)	25	0.525	73.5	32.8	0.063	0.455, 0.595	0.482
Dose (μg)							
DOSE-FEN-OP†	950	0.627	45.8	77.6	0.234	0.564, 0.690	<0.001*
DOSE-FEN-NONOP†	450	0.548	89.8	20.9	0.107	0.473, 0.623	0.209
DOSE-FEN-TOTAL†	1550	0.619	62.3	55.2	0.175	0.552, 0.687	0.001*
BIR (μg/h)							
BIR-FEN-OP†	19	0.634	45.6	77.6	0.232	0.570, 0.698	<0.001*
BIR-FEN-NONOP†	8.5	0.557	89.1	23.9	0.130	0.481, 0.633	0.139
BIR-EME	21	0.532	92.7	13.4	0.061	0.458, 0.605	0.398
BIR-FEN-TOTAL†	31	0.626	62.1	56.7	0.188	0.558, 0.693	<0.001*

AUC, area under the curve; BIR, background infusion rate; BIR-EME, BIR for adjuvant antiemetics; CI, confidence interval. Sens., Sensitivity; Spec., Specificity. *, statistical significance at $p < 0.05$. †, Potential variables (Doses and BIRs) were fentanyl equivalent (μg) doses converted for opioids (DOSE-FEN-OP), non-opioid adjuvant analgesics (DOSE-FEN-NONOP), and total analgesics (DOSE-FEN-TOTAL) using the ratios of oxycodone (μg) to fentanyl (100:1), sufentanil (μg) to fentanyl (1:10), ketorolac (mg) to fentanyl (25:100), and nefopam (mg) to fentanyl (20:100), and BIRs were recalculated with these converted doses (BIR-FEN-OP, BIR-FEN-NONOP, and BIR-FEN-TOTAL).

Table S6. Cutoff values of potential variables for requiring rescue antiemetics in patients with high PPI grade (n = 800)

Potential variables	Cutoff value	AUC	Sens. (%)	Spec. (%)	Youden index	95% CI	p value
PCA setting							
BIR (1 mL/h)	1.75	0.541	99.5	8.7	0.082	0.413, 0.668	0.530
Bolus volume (1 mL)	0.5	0.491	89.1	13.0	0.021	0.373, 0.610	0.886
Lockout interval (min)	12.5	0.522	85.1	21.7	0.068	0.399, 0.646	0.721
Dose (μg)							
DOSE-FEN-OP†	850	0.614	61.4	60.9	0.223	0.481, 0.746	0.093
DOSE-FEN-NONOP†	700	0.629	63.4	60.9	0.243	0.510, 0.748	0.033*
DOSE-FEN-TOTAL†	1450	0.670	74.8	60.9	0.357	0.541, 0.798	0.010*
BIR (μg/h)							
BIR-FEN-OP†	17	0.641	61.3	65.2	0.265	0.507, 0.774	0.039*
BIR-FEN-NONOP†	14	0.660	63.2	65.2	0.284	0.541, 0.778	0.008*
BIR-EME	21	0.539	89.8	17.4	0.072	0.412, 0.665	0.549
BIR-FEN-TOTAL†	29	0.700	74.5	65.2	0.397	0.575, 0.826	0.002*

AUC, area under the curve; BIR, background infusion rate; BIR-EM, BIR for adjuvant antiemetics; CI, confidence interval. Sens., Sensitivity; Spec., Specificity. *, statistical significance at $p < 0.05$. †, Potential variables (Doses and BIRs) were fentanyl equivalent (μg) doses converted for opioids (DOSE-FEN-OP), non-opioid adjuvant analgesics (DOSE-FEN-NONOP), and total analgesics (DOSE-FEN-TOTAL) using the ratios of oxycodone (μg) to fentanyl (100:1), sufentanil (μg) to fentanyl (1:10), ketorolac (mg) to fentanyl (25:100), and nefopam (mg) to fentanyl (20:100), and BIRs were recalculated with these converted doses (BIR-FEN-OP, BIR-FEN-NONOP, and BIR-FEN-TOTAL).
